# Role of catalytic iron and oxidative stress in nitrofen-induced congenital diaphragmatic hernia and its amelioration by Saireito (TJ-114)

**DOI:** 10.3164/jcbn.17-17

**Published:** 2017-09-05

**Authors:** Shima Hirako, Hiroyuki Tsuda, Fumiya Ito, Yasumasa Okazaki, Tasuku Hirayama, Hideko Nagasawa, Tomoko Nakano, Kenji Imai, Tomomi Kotani, Fumitaka Kikkawa, Shinya Toyokuni

**Affiliations:** 1 Department of Pathology and Biological Responses, Nagoya University Graduate School of Medicine, 65 Tsurumai-cho, Showa-ku, Nagoya 466-8550, Japan; 2 Department of Gynecology and Obstetrics, Nagoya University Graduate School of Medicine, 65 Tsurumai-cho, Showa-ku, Nagoya 466-8550, Japan; 3 Laboratory of Pharmaceutical and Medicinal Chemistry, Gifu Pharmaceutical University, Gifu 501-1196, Japan; 4 Sydney Medical School, The University of Sydney, Sydney, NSW 2006, Australia

**Keywords:** congenital diaphragmatic hernia, nitrofen, Saireito

## Abstract

Congenital diaphragmatic hernia (CDH) is a life-threatening neonatal disease that leads to lung hypoplasia and pulmonary hypertension. We recently found that maternal prenatal administration of Saireito (TJ-114) ameliorates fetal CDH in a nitrofen-induced rat model. Here, we studied the role of iron and oxidative stress in neonates of this model and in lung fibroblasts IMR90-SV in association with nitrofen and Saireito. We observed increased immunostaining of 8-hydroxy-2'-deoxyguanosine in the lungs of neonates with CDH, which was ameliorated by maternal Saireito intake. Pulmonary *transferrin receptor* expression was significantly decreased in both CDH and CDH after Saireito in comparison to normal controls, indicating functional lung immaturity, whereas catalytic Fe(II) and pulmonary *DMT1/ferroportin* expression remained constant among the three groups. Saireito revealed a dose-dependent scavenging capacity with electron spin resonance spin trapping *in vitro* against hydroxyl radicals but not against superoxide. Finally, nitrofen revealed dose-dependent cytotoxicity to IMR90-SV cells, accompanied by an increase in oxidative stress, as seen by 5(6)-chloromethyl-2',7'-dichlorodihydrofluorescein diacetate and catalytic Fe(II). Saireito ameliorated all of these in IMR90-SV cells. In conclusion, catalytic Fe(II)-dependent oxidative stress by nitrofen may be the pathogenic cause of CDH, and the antioxidative activity of Saireito is at least partially responsible for improving nitrofen-induced CDH.

## Introduction

Congenital diaphragmatic hernia (CDH) is a developmental anomaly characterized by the failure of regions of the diaphragmatic musculature to form during embryogenesis, which allows developing abdominal contents to invade the thoracic cavity, occupying space normally reserved to accommodate the growing lung. As a result, newborns with CDH (2.4 per 10,000 births) suffer from a combination of pulmonary hypoplasia, pulmonary hypertension and surfactant deficiency.^(1,2)^ Despite the progress in prenatal diagnosis, prognostic marker and postnatal care,^(3)^ CDH induces a high rate of mortality (approximately 20~30%) with a high medical cost.^(4,5)^ Therefore, antenatal pharmaceutical intervention is highly anticipated after the diagnosis *in utero* in humans.

A rat CDH model induced by a herbicide, nitrofen (2,4-dichlorophenyl-*p*-nitrophenyl ether), was established in 1971 and is well known for the presence of lung hypoplasia and persistent pulmonary hypertension,^(6)^ similar to those observed in human CDH patients.^(7–10)^ In this model, pregnant Sprague-Dawley rats are exposed to nitrofen prior to diaphragm formation during gestation (~embryonic day 9, E9), leading to a high incidence of CDH. While the molecular mechanism of nitrofen-induced CDH is elusive, including altered retinoid and thyroid signalling pathways,^(11–13)^ an observation of hypoplastic lung in non-CDH fetuses suggests a direct action of nitrofen on fetal lungs as well.^(14)^

Here, oxidative stress may be responsible for the pathological action of nitrofen in CDH.^(15,16)^ Antioxidants such as vitamin E and vitamin C accelerate the growth of hypoplastic lungs *in vitro* and *in vivo*.^(17–19)^ Alternatively, NAD(P)H oxidase activity and *Nox1/Nox2* mRNA levels were increased in the lungs of fetuses with CDH, whereas *SOD1*, *SOD2* and *catalase* mRNA levels were decreased.^(20)^ Because iron distribution and metabolism are central to the initiation of oxidative stress (Fenton reaction),^(21–23)^ we decided to analyse the nitrofen-induced CDH model from the viewpoint of iron, especially catalytic Fe(II), which can be visualized with recently developed fluorescent probes, RhoNox-1 and HM-RhoNoxM.^(24–28)^

As a candidate prenatal drug for the diagnosis of CDH *in utero*, Saireito (TJ-114) is a Japanese herbal medicine composed of 12 distinct crude drugs in fixed proportions: 7.0 g of *Bupleurum* root, 5.0 g of *Pinellia* tuber, 5.0 g of *Alisma* rhizome, 3.0 g of *Scutellaria* root, 3.0 g of *ginseng*, 3.0 g of *Poria* sclerotium, 3.0 g of *Polyporus* sclerotium, 3.0 g of *Atractylodes lancea* rhizome, 3.0 g of *jujube*, 2.0 g of *Glycyrrhiza*, 2.0 g of *cinnamon *bark, and 1.0 g of *ginger*. Saireito has been used in Japan to treat patients with edema and proteinuria during pregnancy. Saireito affects the hypothalamic-pituitary-adrenal axis, stimulating secretion of the corticotrophin releasing factor, followed by hypothalamic ACTH secretion resulting in accelerating the secretion of glucocorticoids with a reduced negative feedback effect.^(29)^ Saireito thus far ameliorated mesangioproliferative glomerulonephritis and peritoneal fibrosis in rats.^(30,31)^ We recently showed in the rat CDH model that antenatal Saireito increased the lung volume and alveolarization and respiratory functions while decreasing *endothelin-1* expression and vascular remodelling, thus lowering the incidence of CDH.^(32)^ Here, we focused on iron metabolism and oxidative stress in nitrofen-induced rat CDH and its prevention by Saireito.

## Materials and Methods

### Chemicals

Nitrofen (2,4-dichlorophenyl-*p*-nitrophenyl ether; Sigma-Aldrich, St. Louis, MO; 100 mg) was dissolved in 1 ml of olive oil (Wako, Osaka, Japan) for animal experiments and was dissolved in dimethyl sulfoxide (Wako) to 100 mM for further dilution in cell experiments. Diethylenetriamine-*N*,*N*,*N'*,*N''*,*N''*-pentaacetic acid (DTPA) was from Dojindo (Kumamoto, Japan). FeSO_4_ and hypoxanthine was from Wako, xanthine oxidase was from Sigma-Aldrich and 5,5-dimethyl-1-pyrroline-*N*-oxide (DMPO) was from Labotec (Tokyo, Japan). All the chemicals were of analytical grade.

### Animals

The animal experimental committee of Nagoya University Graduate School of Medicine approved the following experiments (No. 28241). Timed pregnant female Sprague-Dawley rats were purchased from Chubu Kagaku Shizai (Nagoya, Japan). All rats were maintained on a 12 h light/12 h dark cycle at 22–24°C in specific pathogen-free conditions.

### Experimental design of the animal model

Rats received an oral administration of 100 mg nitrofen on day 9 of pregnancy (E9) as previously described and control animals received 1 ml of olive oil.^(10)^ The incidence of CDH varies depending on the timing of nitrofen administration and is approximately 40~60%.^(33,34)^ Nitrofen-exposed pregnant rats were randomized to two groups: nitrofen + olive oil or nitrofen + Saireito treatment. Saireito (Tsumura & Co., Tokyo, Japan) (2,000 mg/kg/day) was dispersed in 3 ml of PBS and orally administered to the nitrofen-treated pregnant rats from E10 to E20. At term (E21) fetuses were collected by Caesarean section, followed by thoraco-laparotomy to inspect the diaphragm. The lungs were harvested and either fixed in neutral buffered 10% formalin or frozen at −80°C for the subsequent analysis. Because not all the fetuses developed CDH, only those that developed CDH were included in the present analysis. To determine the effect of Saireito, we defined the three groups as follows: normal untreated rats (N), nitrofen-induced CDH (CDH) and nitrofen-induced CDH with Saireito treatment (CDH + S).

### Immunohistochemistry

We used phosphate-buffered 10% formalin-fixed, paraffin-embedded tissue sections of the lung and performed immunohistochemical analysis as described.^(35)^ The antibodies used were as follows: 8-hydroxy-2'-deoxyguanoisine (8-OHdG; N45.1, 5 µg/ml; JICA, Fukuroi, Japan); divalent metal transporter (DMT1; rabbit polyclonal, 1:100; Santa-Cruz, Dallas, TX); transferrin receptor (mouse monoclonal, 13-6800; 2 µg/ml; Zymed, San Francisco, CA) and ferroportin (rabbit polyclonal, 1 µg/ml; Abcam, Cambridge, UK). For the quantification of 8-OHdG immunohistochemical analysis, the percentage of positive cells was scored as: 1, 0–5%; 2, 5–25%; 3, 25–50%; 4, 50–75% and 5, >75% as described (*n* = 11).^(36,37)^ Measurements were determined at 100× magnification in 10 randomly selected fields. A rabbit monoclonal antibody against caspase 3 was from Cell Signaling (#9664, Danvers, MA).

### Western blot analysis

Western blot analysis was performed as described.^(38)^ Briefly, lung tissues (*n* = 3 fetuses per group) were homogenated in RIPA lysis buffer (Thermo Fisher Scientific Inc.; Waltham, MA) containing inhibitor cocktail tablets (Roche Diagnostics GmbH, Mannheim, Germany). They were then centrifuged at 250 × *g* at 4°C for 10 min to obtain supernatants. A total of 30 µg protein per lane was loaded. The polyvinylidine difluoride membrane was incubated with primary antibody (transferrin receptor antibody, 1:250, and β-actin antibody, 1:5,000). Anti-mouse IgG HRP-linked antibody (#7076S, 1:1,000; Cell Signalling Technology, Danvers, MA) was used as a secondary antibody.

### Measurement of electron spin resonance (ESR) spin trapping

We obtained ESR spin trapping spectra as described,^(39)^ using a JES-FR30 Free Radical Monitor (JEOL RESONANCE, Tokyo, Japan). The reaction samples were mixed and aspirated to a disposable flat cell (RDC-60; Flashpoint, Tokyo, Japan). The experimental condition was as follows: power, 4 mW; modulation width, 0.04 mT; sweep time, 2 min; sweep width, 15 mT; time constant 0.1 s. The standard reaction mixture (200 µl in a total volume) for hydroxyl radicals consisted of 100 µM DTPA in PBS (120 µl), 50 µM FeSO_4_ (20 µl), DMPO (1:1,000 dilution; 20 µl), 1 mM H_2_O_2_ (20 µl) and sample/PBS (20 µl). Saireito (100 µg/ml) dispersed in PBS was incubated at 40°C for 30 min and passed through a sterile syringe filter with a 0.22-µm pore (Millipore, Darmstadt, Germany). Saireito in a different dilution (0.05, 0.1, 0.2, 1.0 and 2.0 µg/ml) was used instead of 20 µl of PBS for ESR evaluation. Sho-saikoto and Goreisan (Tsumura), two components of Saireito and constituting a different herbal medicine, respectively, were used in the same manner as Saireito for the ESR experiments. ESR spectra (1:2:2:1) of DMPO/OH radical adducts were detected. To measure the superoxide scavenging activity by ESR, the standard reaction mixture consisted of PBS (100 µl), 4 mM DTPA (20 µl), 2 mM hypoxanthine (20 µl), xanthine oxidase (20 µl), DMPO (×1) 1 µl and PBS (20 µl) were used. The ESR signal was recorded 1 min after mixing all the components. The ESR spectrum of each radial adduct was detected, and the peak height of the ESR signal was measured and calculated as the % of control. The measurements were performed in triplicate.

### Cell culture

The human lung fibroblast cell line IMR90-SV (RIKEN CELL BANK, Saitama, Japan) was maintained in RPMI (Sigma-Aldrich) supplemented with 10% fetal calf serum, penicillin (100 U/ml) and streptomycin (100 µg/ml) at 37°C under a 5% CO_2_ atmosphere. Saireito was dispersed either in RPMI or PBS at 40°C to 100 µg/ml solution, followed by passing through a sterile syringe filter with a 0.22-µm pore size and was used.

### Cell viability assay

IMR90-SV cells seeded at a density of 1.0 × 10^4^/ml in a 96-well culture plate were incubated for 18 h at 37°C, and 200 µl of nitrofen (0.1, 0.5, 1.0 and 5.0 mM), nitrofen in the presence of 100 µg/ml Saireito or fresh complete medium was added after washing. After 72 h, 10 µl of the 12 mM MTS [3-(4,5-dimethylthiazol-2-*yl*)-5-(3-carboxymethoxyphenyl)-2-(4-sulfophenyl)-2H-tetrazolium, inner salt] (Promega, Tokyo, Japan) solution was added to each well and incubated at 37°C for 1 h, followed by reading absorbance at 450 nm (Vient 808; DS pharma Biomedical Co., Ltd., Osaka, Japan). Each experimental condition was performed in 5 wells and was repeated 5 times.

### Detection of oxidative stress

IMR90 cells at a density of 2 × 10^4^ cells per well were seeded in a black culture plate and incubated for 18 h. 5-(and-6)-Chloromethyl-2',7'-dichlorodihydrofluorescein diacetate acetyl ester (CM-H_2_DCFDA; Molecular Probes Invitrogen, Carlsbad, CA) was added (2 µl of 5 µM) to each well and incubated for 30 min at 37°C. The medium was removed and replaced by 200 µl nitrofen (0.1, 1.0 and 5.0 mM) diluted in PBS, nitrofen (0.1, 1.0 and 5.0 mM) with 100 µg/ml Saireito diluted in PBS or control PBS. After 1 h, fluorescence was measured with a microplate reader (Mithras LB 940; Berthold Technologies GmbH & Co., Bad Wildbad, Germany) after excitation at 485 nm and emission at 535 nm. The experiments were performed in duplicate and repeated 5 times.

### Analysis of catalytic iron

HMRhoNox-M and RhoNox-1 were dissolved in dimethyl sulfoxide to 10 mM solution.^(28)^ Lung frozen sections at 8-µm thickness were prepared with a cryostat on MAS-GP type A glass slides (Matsunami, Osaka, Japan), which, after air drying for 5 min, were fixed with 20% neutral formalin in methanol for 3 min and washed with PBS three times. Thereafter, 100 µl of 10 µM RhoNox-1 was directly applied on the slides, which were incubated for 60 min at 37°C in a dark chamber, counterstained with Hoechst 33342 (Thermo Fisher) and observed with a fluorescence microscope (BZ9000; Keyence, Osaka, Japan).

IMR90-SV cells (1.0 × 10^4^/ml) were seeded on 35-mm culture dish and incubated for 18 h at 37°C. Then, the media were removed and 500 µl of either 100 µM nitrofen diluted with RPMI, 100 µM nitrofen with 100 µg/ml Saireito diluted in RPMI or fresh culture control medium was added. After 24 h, 500 µl of 10 µM HMRhoNox-M was added and incubated for 60 min at 37°C in a dark chamber, counterstained with Hoechst 33342 and observed with fluorescence microscope. Fluorescence intensity was quantified with ImageJ (https://imagej.nih.gov/ij/). The experiments were repeated 4 times.

### Statistical analysis

Statistical analyses were performed with R and Excel for Windows 2010 (Microsoft Corp., Redmond, WA). Values were expressed as the means ± SEM and the normality of the data was assessed with the Shapiro-Wilk test. Student’s *t* test or the Mann-Whitney *U* test was performed to assess differences between the means. Multiple group comparisons were performed using Tukey’s test. Statistical significance was defined at *p*<0.05.

## Results

### Catalytic Fe(II)-independent pulmonary oxidative stress in postnatal CDH is reversed by prenatal Saireito

**Fig. 1:**
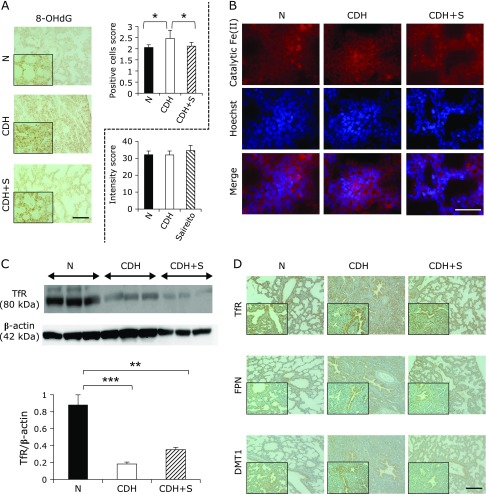
Oxidative stress in the newborn lung of the rat CDH model with an immature iron transport system. (A) Immunohistochemical analysis of 8-OHdG in the newborn lungs. N, normal untreated; CDH, nitrofen-induced congenital diaphragmatic hernia; CDH + S, nitrofen-induced CDH with Saireito treatment. Alveolar epithelial cells in CDH showed more 8-OHdG positive cells than in the N group, which was ameliorated by Saireito (bar = 200 µm; inset 100 µm; means ± SEM, *n* = 11; *p<0.05, Mann-Whitney *U* test). (B) No difference in catalytic Fe(II) with RhoNox-1 staining. Hoechst, Hoechst 33342 nuclear staining (bar = 50 µm). (C, D) Analysis of iron transporters by Western blot analysis and immunohistochemistry. Lungs of CDH and CDH with Saireito treatment presented functional immaturity in iron transport. CDH lungs showed low air content. TfR, transferrin receptor; FPN, ferroportin; DMT1, divalent metal transporter 1 (bar = 200 µm; means ± SEM, *n* = 3; **p<0.01, ***p<0.001). Refer to Materials and Methods and Results sections for details.

8-OHdG levels by immunohistochemistry were significantly higher in CDH lung alveolar cells than in normal counterparts, which were ameliorated by the prenatal administration of Saireito to pregnant rats (Fig. 1A; CDH 2.46 ± 0.10 vs normal 2.06 ± 0.037, *p* = 0.02; CDH + Saireito (S) 2.11 ± 0.050 vs CDH; *n* = 3, *p* = 0.04). However, there were neither significant differences in the amounts of catalytic Fe(II) among the three groups (Fig. 1B) and nor did we observe death of alveolar lining cells, including apoptosis analysed by caspase 3 immunostaining (data not shown).

### Low expression of transferrin receptor in the CDH lung irrespective of Saireito administration

The expression of transferrin receptor (TfR) protein was significantly decreased in the lungs of CDH with Western blot analysis and densitometry analysis, irrespective of Saireito administration (Fig. 1C). Immunohistochemical analysis of TfR revealed low levels of TfR in the CDH lung throughout the entire area, whereas CDH + S showed low levels of TfR, especially in alveolar lining cells. Regarding divalent metal transporter 1 (DMT1; Slc11A2) and ferroportin (FPN; Slc40A1), there was no significant difference among the three groups with immunohistochemistry (Fig. 1D).

### Saireito scavenges hydroxyl radicals but not superoxides

**Fig. 2:**
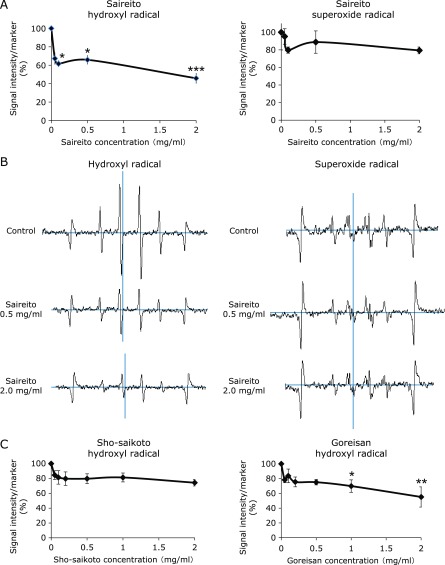
Effects of the water extract of Saireito on the formation of hydroxyl radicals (Fenton reaction) and superoxide radicals with electron spin resonance (ESR) spin trapping analysis. (A) Dose-dependent scavenging of hydroxyl radicals by the water extract of Saireito (means ± SEM, *n* = 5; *p<0.05, ***p<0.001), which was not observed for superoxide radicals. (B) Typical ESR spin trapping signals. Signals of both sides are Mn(3) and Mn(4) markers as controls. (C) Scavenging of hydroxyl radicals by the water extract of Goreisan, but not of Sho-saikoto (means ± SEM, *n* = 5; *p<0.05, **p<0.01).

We obtained ESR spin trapping spectra of DMPO/OH and DMPO/OOH radical adducts and studied the effects of water-soluble extracts of Saireito. Saireito dose-dependently inhibited the signal of hydroxyl radicals but not superoxide radicals (Fig. 2A and B). Then, we analysed which of the two components of Saireito, Sho-saikoto or Goreisan, would play a major role, and we found that Goreisan works better than Sho-saikoto to scavenge the hydroxyl radicals (Fig. 2C).

### Cytotoxicity of nitrofen to lung fibroblasts is associated with oxidative stress and an increase in cytoplasmic catalytic Fe(II)

**Fig. 3:**
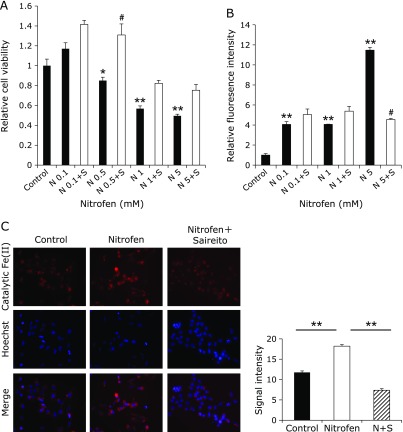
Cytotoxicity of nitrofen to IMR90-SV lung fibroblasts evaluated by oxidative stress and catalytic Fe(II). (A) Dose-dependent cytotoxicity of nitrofen, which was ameliorated by the water extract of Saireito. N, nitrofen; N + S, nitrofen with Saireito treatment. (means ± SEM, *n* = 5; *p<0.05, **p<0.001 vs Control; ^#^*p*<0.05 vs paired N group). (B) Evaluation of oxidative stress seen by CM-H2DCFDA (means ± SEM, *n* = 5). (C) Assessment of catalytic Fe(II) with HMRhoNox-M. Increase in catalytic Fe(II) with nitrofen treatment and its amelioration by the water extract of Saireito (means ± SEM, *n* = 4). Refer to Materials and Methods and Results sections for details.

The addition of nitrofen to the media resulted in a dose-dependent decrease in the viability of lung fibroblasts, which was ameliorated by the simultaneous addition of the water extract of Saireito (Fig. 3A). The cytotoxicity was proportionally associated with oxidative stress as evaluated by CM-H2DCFDA and cytoplasmic catalytic Fe(II), which was also ameliorated by the addition of Saireito (Fig. 3B).

## Discussion

The administration of nitrofen to pregnant rats is an established model of CDH.^(14)^ In the present study, for the first time, we showed the association of catalytic Fe(II) in the cytotoxicity of nitrofen by the use of a novel fluorescent probe HM-RhoNox-M.^(27,28)^ Because catalytic Fe(II) initiates the Fenton reaction in the presence of certain concentrations of H_2_O_2_,^(15)^ the clinical use of iron chelating agents, such as desferal and defearasirox, may be considered. However, it is impossible in human clinical situations for the following reasons. First, it is not easy to determine the critical periods for CDH generation in human pregnancies, and second, pregnant mothers require large quantities of iron for fetal growth. In this sense, avoiding possible teratogens for CDH would be the best strategy for the prevention of CDH.

We thus considered a strategy to ameliorate CDH when diagnosed *in utero*.^(32)^ Based on the previous report that non-CDH affected lungs in the newborns of nitrofen-induced CDH models are also hypoplastic, we hypothesized that there are direct effects of nitrofen to the lung in addition to indirect effects on the retinoid and thyroid signalling pathways as reported previously.^(11–13)^ Saireito, a Japanese herbal drug, has been registered and used for pregnant patients in Japan to reduce edema. We evaluated the scavenging activity of Saireito against superoxides and hydroxyl radicals and found that Saireito scavenges only hydroxyl radicals, which indicates that the water extract of Saireito decreases the effective concentration of either catalytic Fe(II) or H_2_O_2_. Indeed, the water extract of Saireito was also effective in the *in vitro *system using lung fibroblasts (IMR90).

We thereafter studied the neonatal lungs in the CDH model. The alveolar structure was immature in the CDH model, concomitant with oxidative stress, as demonstrated by the quantities of 8-OHdG. Oxidative stress in the neonatal lung was significantly ameliorated after Saireito administration *in utero* in the CDH model. However, the quantities of catalytic Fe(II) were not altered among the three groups analysed, and iron transporters (transferrin receptor, DMT1 and ferroportin) revealed significantly lower expression levels in the CDH/Saireito as well as CDH groups, which indicated that the amelioration of lung immaturity is not perfect, even after treatment with Saireito* in utero.* In total, the neonatal pulmonary oxidative stress we observed is probably associated with respiratory distress at birth due to lung immaturity and not with the direct effect of nitrofen.

Saireito is a combination of two different herbal drugs, Goreisan and Sho-saikoto. Cinnamon in Goreisan and Baicalin in Sho-saikoto have also been reported to inhibit the Fenton reaction catalysed by copper or iron.^(40,41)^ These may be the chemicals responsible for the effectiveness of Saireito. In ESR studies, Goreisan played a major role.

There are several limitations to the present study. As a prodrug, Saireito contains water-insoluble components, which results in increasing activity after digestion and absorption. In this sense, our study showed only some of the properties of Saireito, and could not demonstrate this particular action, especially in our *in vitro* experiments. Another limitation is that we did not analyse the fetuses immediately after nitrofen administration to pregnant rats due to the technical difficulty of this procedure. Further studies are warranted with novel techniques.

In conclusion, the maternal administration of Saireito in the nitrofen-induced CDH model ameliorated the oxidative stress in the affected lungs with CDH, which were still immature in function. The induction of oxidative stress by nitrofen in fibroblasts was associated with increased catalytic Fe(II), and the role of Saireito may be explained at least partially by the scavenging activity of the Fenton reaction.

## Abbreviations

CDH: congenital diaphragmatic hernia
DMPO: 5,5-dimethyl-1-pyrroline-*N*-oxide
DMT1: divalent metal transporter 1 (Slc11A2)
DTPA: diethylenetriamine-*N*,*N*,*N'*,*N''*,*N''*-pentaacetic acid
E9: embryonic day 9
ESR: electron spin resonance
FPN: ferroportin
MTS: [3-(4,5-dimethylthiazol-2-*yl*)-5-(3-carboxymethoxyphenyl)-2-(4-sulfophenyl)-2H-tetrazolium, inner salt]
Nitrofen: 2,4-dichlorophenyl-*p*-nitrophenyl ether
8-OHdG: 8-hydroxy-2'-deoxyguanoisine
PBS: phosphate-buffered saline
S: Saireito
TfR: transferrin receptor
